# Changing Epidemiology of Carbapenemases Among Carbapenem-Resistant Enterobacterales in a Greek Tertiary Care Hospital in Athens, 2020 to 2023 †

**DOI:** 10.3390/antibiotics14030239

**Published:** 2025-02-26

**Authors:** Vasiliki Koumaki, Eleni Voudanta, Aikaterini Michelaki, Maria Orfanidou, Eleni Vagiakou, Georgia Vrioni, Athanassios Tsakris

**Affiliations:** 1 Department of Microbiology, Medical School, National and Kapodistrian University of Athens, 115 27 Athens, Greece; elvoudanta@med.uoa.gr (E.V.); gvrioni@med.uoa.gr (G.V.); atsakris@med.uoa.gr (A.T.); 2 Department of Microbiology, General Hospital G. Gennimatas, 115 27 Athens, Greece; kmichelaki@yahoo.gr (A.M.); mariacorf@gmail.com (M.O.); elenivagiakou@gmail.com (E.V.)

**Keywords:** antimicrobial resistance, carbapenemase-producing Enterobacterales, *Klebsiella pneumoniae*, epidemiology, Gram-negative bacteria, Greece

## Abstract

**Background:** Carbapenemase-producing Enterobacterales (CPEs) represent a significant global health threat, particularly in the context of nosocomial infections. The current study constitutes a retrospective epidemiological survey that aimed to provide updated data on the prevalence and characteristics of carbapenemases among carbapenem-resistant Enterobacterales (CREs) in a Greek tertiary hospital in Athens during and after the COVID-19 pandemic. **Results**: A total of 2021 non-duplicate CPE clinical isolates were detected. A significant increase in the number of carbapenemase-positive Enterobacterales was revealed during the study period (*p* < 0.05). KPC remained the predominant carbapenemase type through all four years of the survey, representing 40.7%, 39.9%, 53.5%, and 45.7% of the CPE isolates, respectively. However, a rapid transition from VIM to NDM metal-β-lactamase types was revealed, changing the epidemiological image of carbapenemases in the hospital setting. Notably, among the CPEs, antimicrobial resistance rates were significantly raised in the post-COVID-19 period (2022 and 2023) compared to the first study year (2020) for almost all the tested antibiotics, including those characterized as last-resort antibiotics. **Methods**: CREs were identified and subjected to screening for the five most prevalent carbapenemase genes [*Klebsiella pneumoniae* carbapenemase (KPC), Verona integron-borne metallo-β-lactamase (VIM), New Delhi metallo-β-lactamase (NDM), imipenemase (IMP), and oxacillin-hydrolyzing (OXA-48)] using a lateral flow immunoassay, and the CREs recovered from blood cultures were analyzed using a FilmArray system. Their clinical and epidemiological characteristics, as well as their antimicrobial susceptibility profiles, were also subjected to analysis **Conclusions**: Given this alarming situation, which is exacerbated by the limited treatment options, the development of new, effective antimicrobial agents is needed. The continued monitoring of the changing epidemiology of carbapenemases is also imperative in order to undertake rational public health interventions.

## 1. Introduction

Carbapenem-resistant Enterobacterales (CREs) constitute a serious challenge to the antimicrobial therapy of hospital-acquired infections due to limited treatment options, as these organisms possess several mechanisms of antimicrobial resistance [1]. Among the CREs, the most predominant mechanism of carbapenem resistance is the production of carbapenemases, with the most prevalent type being class A β-lactamase *Klebsiella pneumoniae* carbapenemase (KPC). This carbapenemase prevails in Europe, mostly in Mediterranean countries, as well as in North, Central, and South America [2,3]. Other enzymatic sources of carbapenem resistance comprise the class B metallo-β-lactamases (MBLs), which include Verona integron-borne metallo-β-lactamase (VIM), New Delhi metallo-β-lactamase (NDM), and imipenemase (IMP). NDM constitutes an endemic carbapenemase in India, with a significant number of cases in Asia/Pacific region and Europe. VIM-type carbapenemases are detected mostly in Middle East Africa, while IMP enzymes are endemic in Japan and Taiwan, mainly appearing in Asia and Europe. Finally, class D carbapenemases, including OXA-48-like enzymes, are mostly detected in Europe, the Middle East, Africa, and Asia [4,5,6]. Acquisition of the above carbapenemase genes in CRE strains often co-localizes with other resistance genes on mobile genetic elements, complicating treatment strategies. Thus, resistance to carbapenems often extends to aminoglycosides and fluoroquinolones, leaving clinicians with only a few possibly effective antibiotics, such as colistin, fosfomycin, tigecycline, and in some cases, aztreonam [1].

In Greece, both VIM and KPC enzymes have been endemic for almost three decades. Since the early 2000s, there has been an epidemic of polyclonal VIM-producing *K. pneumoniae*, followed by a monoclonal outbreak of KPC-2-producing *K. pneumoniae* [7]. The first NDM-1-producing *K. pneumoniae* isolate was reported in 2011 in the University Hospital of Ioannina, Epirus [8], while OXA-48 was firstly described in 2012 [9] and still remains rarely isolated. Until recently, KPC was reported as the predominant carbapenemase, followed by NDM [10].

However, an alarming epidemiological change has occurred in Greece in recent years, characterized by a rapid increase in the number of cases of carbapenem-resistant Enterobacteriaceae (CRE), causing both nosocomial outbreaks and sporadic cases of infection [10,11]. Remarkably, according to the most recent annual surveillance report from the European Centre for Disease Prevention and Control and World Health Organization (2021), Greece has one of the highest rates of carbapenem-resistant gram-negative bacteria in Europe [12].

Until now, sporadic updated epidemiological data have been published concerning carbapenem-resistant enterobacterial strains in Greece [10,11,13,14,15,16]. Taking into account that the COVID-19 pandemic has had an impact on the epidemiology of CRE globally, the current study aimed firstly to record the current epidemiology of carbapenemases among CRE clinical isolates and secondly to assess the antimicrobial resistance profiles of CPE strains in one of the largest tertiary care hospitals in Athens, Greece, receiving many referrals from other hospitals from all over the country within the 2020–2023 timeframe. In this work, the study presented in ESCMID Global, Congress of the European Society of Clinical Microbiology and Infectious Diseases, has been expanded [2,3].

## 2. Results

### 2.1. Clinical Isolates of the Study

A total of 2,021 CPEs were identified among 2,038 CRE single-patient clinical isolates recovered during the study period in the clinical lab of the tertiary care general hospital “Georgios Gennimatas”, responsible for infections among patients attending various departments of the hospital. The number of patients infected with CPE isolates each year is detailed in Table 1. Throughout most years, the percentage of male patients was higher, except for the year 2022, when the distribution was 47% for male and 53% for female.

**Figure 1:**
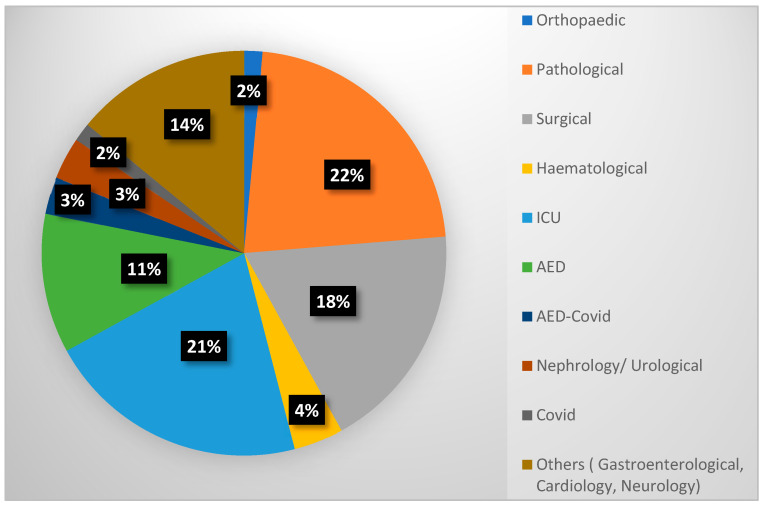
Distribution of carbapenemase-producing isolates by hospital department.

The studied population was gathered from various medical departments of the hospital, including Orthopedic, Pathological, Surgical, Hematology, intensive care unit (ICU), Accident and Emergency Department (AED), Accident and Emergency Department—COVID, Nephrology/Urology, and others. Notably, the Pathological department reported the highest percentage of carbapenemase-producing isolates, at 22.2%, followed by the ICU with 21%. The Surgical department ranked third, with a total percentage of 18.3% across all years (Figure 1).

Clinical isolates were collected from various sources, including blood, urine, bronchial secretions, wound specimens, and multiple sample types. Across all the years studied, urine samples were the most common, comprising 39.6%, 39.5%, 47.5%, and 44.9% of the total isolates. Bloodstream infections accounted for 13.9%, 17%, 15.2%, and 7.6%, respectively. Multiple infections were observed at rates of 17.2%, 15%, 12.5%, and 25.4%, while bronchial secretions represented 9.1%, 13.8%, 11.3%, and 3.9% of the total specimens. Wound specimens were present in percentages of 13.1%, 8.6%, 4.7%, and 0%, and central venous catheter (CVC) samples accounted for 7.1%, 5.9%, 3.4%, and 3.9% of the total specimens each year, respectively (Table 2).

### 2.2. Carbapenemase-Producing Isolates During 2020–2023

A continuous increase in the total number of carbapenemase-producing isolates throughout the studied period was observed (396 isolates in total in 2020 to 633 isolates in 2023; Table 3). Spearman correlation analysis suggested that there is a statistically significant positive correlation between the years and the total number of CPE infections per year (*p* < 0.01). As expected, *K. pneumoniae* constituted the main pathogen among Enterobacterales, with percentages of 89.6%, 94.1%, 89.7%, and 90.2% for each year studied. For the years 2020, 2021, 2022, and 2023, *E. coli* was detected in percentages of 2.8%, 1.8%, 1.1%, and 2.7% respectively. *Enterobacter* spp. was detected at a rate of 3%, 1.4%, 1.8%, and 1.1% for the given time frame. As for *Providencia stuartii* strains, rates of 1.3%, 1.1%, 4.2%, and 3.3% were observed in each year, respectively, while *Proteus mirabilis* strains fluctuated between 2.3% and 1.6% in the period between 2020 and 2023. Table 4 presents the percentages of Enterobacterales in the ICU department during the survey. Enterobacterales in the ICU accounted for 22.7%, 29.3%, 19.2%, and 15.3%, respectively. The most frequently pathogen observed in the ICU department was *K. pneumoniae*, followed by *Enterobacter* spp., *E. coli*, *P. mirabilis*, *P. stuartii*, and *Morganella* spp.

### 2.3. Distribution of Carbapenemase Types and Their Combinations

In general, KPC was the predominant carbapenemase type detected among CREs. For the years 2020, 2021, 2022, and 2023, KPC was detected in percentages of 40.7% (161/396), 39.9% (176/441), 53.5% (295/551), and 45.7% (289/633), respectively. Regarding VIM carbapenemase, the percentages were 15.4% (61/396), 8.8% (39/441), 12.9% (71/551), and 11.8% (75/633) during the period 2020 to 2023. NDM was detected in percentages of 16.7% (66/396), 25.6% (113/441), 23.4% (129/551), and 26.4% (167/633) for each year studied, respectively. Regarding OXA-48-like carbapenemases, the rates were 10.6% (42/396), 1.1% (5/441), 0.2% (1/551), and 0.5% (3/663) in the given time frame. No statistically significant difference was observed in the prevalence of KPC and VIM carbapenemases for the years 2020 to 2023 at *p* < 0.05, while for NDM and OXA-48, an increase and decrease, respectively, was observed (*p* < 0.05). Notably, as for the total number of MBLs (NDM and VIM), a statistically significant increase was detected in the given timeframe.

**Figure 2:**
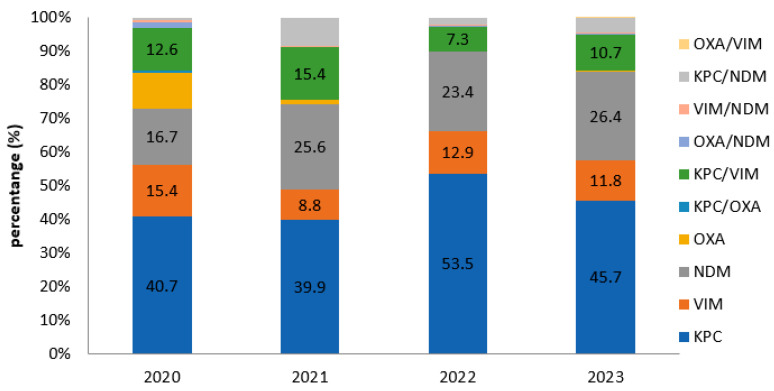
Percentages of carbapenemase type/year.

The most frequent combination among carbapenemases was KPC/VIM, with percentages of 12.6% (50/396), 15.4% (68/441), 7.3% (40/551), and 10.7% (68/633), followed by KPC/NDM at a rate of 0.8% (3/396), 8.4% (37/441), 2% (11/551) and 4.3% (27/633) for each year, respectively. Notably, a bloodstream infection caused by a *K. pneumoniae* was detected in the ICU department in the year 2020, involving a triple combination of KPC/OXA/NDM carbapenemases (Table 5 and Table 6; Figure 2).

**Figure 3:**
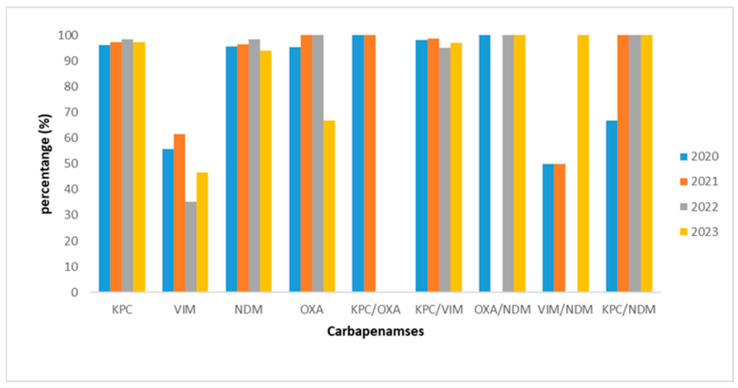
Type of carbapenemase-producing *Klebsiella* spp. during the study period 2020–2023.

The predominant carbapenemase for *K. pneumoniae* was KPC for each year studied (155/355, 171/419, 290/494, 281/571), followed by NDM (63/355, 109/415, 127/494, 157/571) and KPC/NDM (49/355, 67/415, 38/494, 66/571) (Table 6; Figure 3). As for *E. coli*, KPC was predominant for all the years studied, while for *Enterobacter* spp., for the years 2020, 2022, and 2023, VIM (10/12,6/10 and 5/7) was the major carbapenemase. In 2021, NDM (3/6) was detected in half of the cases. As for *P. mirabilis*, *P. stuartii*, and *Serratia* spp., the predominant carbapenemase was VIM for the years 2020–2023. Regarding *Citrobacter* spp., a split between KPC/VIM and KPC/NDM was observed for 2020, while for the year 2022, the split was observed between VIM and VIM/NDM and the unique *Citrobacter* spp., which isolated for the year 2023, was NDM. One *Morganella* spp. strain was isolated in 2020, being VIM positive, while in 2023, two of the three clinical isolates were NDM positive, and the remaining one was a VIM producer (Table 6).

### 2.4. Antimicrobial Resistance Profiles

#### 2.4.1. Total Antimicrobial Resistance Profiles

The antimicrobial resistance patterns for *Klebsiella* spp. from 2020 to 2023, detailing the resistance rates for all the antibiotics examined in this study and their comparison across the periods of 2020–2021, 2021–2022, 2022–2023, and 2020–2023, are illustrated in Table 7. Notably, resistance rates for *Klebsiella* spp. isolates increased significantly in 2022–2023 compared to 2020 for all the tested antibiotics.

Regarding ciprofloxacin and levofloxacin, the resistance rate significantly increased to 94.8%, 97.1%, 97.8%, 98.8% for ciprofloxacin and 94.1%, 96.1%, 97%, 97.5% for levofloxacin for the years 2020–2023, respectively. As for piperacillin/tazobactam, the resistance rate was 92.3%, 99.3%, 99.6%, and 100% for each year, respectively. For the cephalosporins, the resistance rate for cefepime was 88.7%, 97.6%, 97%, and 98.8%, and for ceftazidime, it was 98.3%, 99.8%, 99.8%, and 100% for the years 2020, 2021, 2022, and 2023, respectively, indicating a statistically significant increase throughout the years studied. Resistance to ceftazidime/avibactam was detected in rates of 6.1%, 8.3%, 32.1%, and 49.7%, reflecting the introduction of the new agent into the Greek market as well as the prevalence of the metallo-β-lactamases, since all resistant clinical isolates were positive for metallo-β-lactamases.

As for aminoglycosides, the resistance rates for gentamicin were 43.4%, 72.3%, 73.2%, and 73.7%; for tobramycin, they were 88.7%, 94.5%, 91.8%, and 95.1%; and for amikacin, they were 48.9%, 82.9%, 79.5%, and 81.6% for each year, respectively. For aztreonam, the resistance rates were detected in percentages of and 87.6%, 94%, 95.7%, and 94.9% in the years 2020, 2021, 2022, and 2023, respectively, indicating a statistically significant increase throughout the years studied. As for trimethoprin/sulfamethoxazole, a statistically significant increase was revealed across the years, with percentages of 71.8%, 85.5%, 83.1%, and 88.6%. The nitrofurantoin resistance rate was detected in percentages of 81.3%, 93.9%, 94.9%, and 97.9%, indicating a statistical increase throughout the years studied. As for fosfomycin (per os), the available data were not sufficient for comparison for the years 2020 and 2021. Nevertheless, the rate of antimicrobial resistance, detected as 20.7%, 100%, 81.8%, and 92.3% for each year studied, revealed statistically significant resistance among the years.

Regarding the fosfomycin iv formulation, the resistance rate exhibited a statistically significant increase from 28.3% in 2021 to 59.9% and 75.9% for the years 2022 and 2023, respectively, while no data were available for 2020. As for fosfomycin, per os, no conclusions can be drawn since the sample was limited, and more importantly, there are no breakpoints for *Klebsiella* spp. according to the EUCAST from 2021.

Resistance to colistin was 45.3%, 33.7%, 39.6%, and 43.2% for each year studied, respectively, indicating a fluctuation throughout the years studied. A statistically significant decrease was noted, likely reflecting the restrained use of colistin in the hospital due to nephrotoxicity. The tigecycline resistance rates were 40.7%, 96.7%,95.8% and 95.6% for the years 2020–2023. No statistically significant difference was observed for the year 2022–2023.

All the antibiotics showed a significant increase in resistance from 2020 to 2023. For the majority of the antibiotic agents, no significant changes were observed between 2021–2022 and 2022–2023, except for cefepime, trimethoprin/sulfamethoxazole, colistin, ceftazidime/avibactam, and fosfomycin.

#### 2.4.2. Antimicrobial Resistance Profiles Stratified by Carbapenemase Type

The antibiotic resistance rates of Enterobacterales stratified by carbapenemase type throughout the study period are depicted on Table 8. As for KPC-positive isolates, resistance rates to ceftazidime/avibactam, gentamicin, amikacin, sulfomethoxazole, colistin, and fosfomycin iv were 0%, 62.9%, 75.6%, 68.6%, 34.9%, and 64.8%, respectively. Regarding the NDM-positive isolates, resistance rates to aztreonam, gentamicin, amikacin, sulfomethoxazole/trimethoprim, and colistin were 88.2%, 70.5%, 75.6%, 82.9%, and 54.7%, respectively. Among the VIM-positive isolates, the resistance rates of aztreonam, gentamicin, amikacin, colistin, and fosfomycin iv were 63.4%, 72.8%, 78%, 58%, and 66.2%, respectively. In the OXA-48-positive isolates, the resistance rates to ceftzazidime/avibactam gentamicin, amikacin, sulfomethoxazole/trimethoprim, and colistin were 0%, 82.4%, 17.6%, 36%, and 82%, respectively. Among the KPC/VIM-positive isolates, the resistance rates to colistin and fosfomycin iv were 45.3% and 55.9%. As for the KPC/NDM-positive isolates, the resistance rates to gentamicin, nitrofurantoin, colistin, and fosfomycin iv were 52.6%, 69.2%, 32.5%, and 81.3%. All other antibiotic resistance rates, as stratified by carbapenemase type, were over 85%. An isolate possessing both OXA and VIM-type carbapenemases recovered in 2023 in our hospital exhibited resistance to all antibiotics except for colistin and fosfomycin. The triple combination of KPC/OXA/NDM showed susceptibility only to amikacin and trimethoprin/sulfamethoxazole.

## 3. Discussion

Microbial resistance remains a significant public health concern worldwide. The rise of CREs is steadily increasing, posing a serious challenge to infection treatment for the scientific community. Numerous studies have highlighted a growing prevalence of CRE infection and colonization, underscoring the complex and diverse impacts of the pandemic on antimicrobial resistance patterns [17].

This study aimed to investigate the epidemiology of carbapenemases among CRE clinical strains isolated from a tertiary hospital in Athens, Greece, over the period of 2020 to 2023, and to assess the antimicrobial resistance of these strains. For this reason, a total of 2021 single-patient clinical isolates were examined.

Our results indicate a growing number of carbapenemase-producing isolates for the years 2020–2023 (*p* < 0.05), as seen in Table 3 and Table 4. Among them, *Klebsiella* spp. is the most common Enterobacterales species responsible for hospital-acquired infections, with KPC being the most prevalent carbapenemase throughout the study period. This finding aligns with previous research, which confirms that KPC remains the predominant carbapenemase among *K. pneumoniae* in Greece [10,11,14,15]. Notably, the KPC enzyme remains the most common carbapenemase in Europe and North America, as noted by the ATLAS surveillance program [6].

In accordance with other studies that have shown an increasing trend in NDMs, constituting the predominant mechanism of resistance [10,16], in several regions in Greece, our study indicates a decrease in VIM from 15.4% in 2020 to 11.8% in 2024, with a simultaneously increase in NDM enzymes from 16.7% in 2020 to 26.4% in 2024, resulting in metallo-β-lactamases becoming second in carbapenemase prevalence among CPEs. Notably, NDM and MBLs in general showed a statistically significant increase throughout the study period. According to the ATLAS surveillance program, there seemed to be a gradual increase in NDM globally from 2018 to 2022 [6]. In some regions, such as Latin America, Middle East Africa, and the Asia/Pacific region, NDM constitutes the most prevalent type of carbapenemase during these years among Enterobacterales.

According to published data, OXA-48 constitutes one of the main mechanisms of resistance in Europe, as well as in North Africa and in the Middle East [4,5,6]. A significant dissemination of OXA-48-like carbapenemases was noted in Europe in 2015, with varying global trends observed from 2018 to 2022, indicating a slight increase [6,18]. However, in our study, a gradual decline was noted for OXA-48 during the studied period. This could be attributed to the fact that this study was performed in a single hospital; therefore, this might reflect an epidemiological trend. Moreover, since OXA-48 carbapenemases may hydrolyze weakly active carbapenems, some cases attributed to non-CRE isolates may have been missed [19].

Double or/and triple combinations of carbapenemase genes also constitute a serious concern for global health, emphasizing the need for ongoing surveillance and effective infection control measures to eliminate the spread of pathogens. In our study, the most common combination was KPC/VIM, followed by KPC/NDM. The rest of the combinations appeared rarely. In addition, a triple combination of KPC/OXA/NDM in a *K. pneumoniae* strain isolated from the ICU department, causing a bloodstream infection, showed up in 2020. Notably, the strain was resistant to all antibiotics tested except for amikacin and trimethoprin/sulfamethoxazole (Table 5 and Table 6; Figure 2). To our knowledge, CPEs, which carry double or multiple carbapenemases, represent a rising and concerning phenomenon and have been particularly described during the COVID-19 pandemic [20]. The main species co-producing carbapenemase enzymes is *K. pneumoniae*, which is in accordance with our findings. A total of 28 different carbapenemase combinations have been reported, with the top five combinations being NDM coexistence with OXA, KPC coexistence with NDM, VIM coexistence with OXA, KPC coexistence with VIM, and KPC coexistence with OXA, accounting for approximately 70% of the total number of combinations reported across all continents [21]. In our study, KPC/VIM and KPC/NDM were the most prominent combinations.

In most studies, urinary tract infections (UTIs) are recognized as the most common type of infection associated with CREs [4]. Pintado et al. (2021) also found that urinary infections were the most frequent infections occurring among patients with CPE and COVID-19 [22]. The findings of this survey align with these reports, indicating that the major burden of the CREs falls on UTIs.

As for the antimicrobial patterns of the isolates, the resistance rate of the majority of the antibiotics tested in this survey exhibited a statistically significant increase during 2020–2023. The percentage of resistance for almost all antibiotics approached 100%; however, a few antibiotics, namely gentamicin, fosfomycin, amikacin, and trimethoprim/sulfamethoxazole, exhibited resistance rates that were somewhat lower in 2023, i.e., 73.7%, 75.9%, 81.6%, and 88.65% respectively, indicating that these antibacterials might be considered as last treatment options for such infections.

Aztreonam, a β-lactam antibiotic of the monoamide ring class, is specifically recommended for treating MBL carbapenemase-producing CREs, often in combination with ceftazidime/avibactam [23]. In our study, the resistance rates for aztreonam were 87.6%, 94%, 95.7%, and 94.9%, for the years 2020–2023. The fact that the majority of our strains were resistant to aztreonam as well may indicate the presence of a second gene—for example, ESBL—which indicates an increasing resistance to a broad range of antibiotics. These data suggest that aztreonam/avibactam could offer a solution for the treatment of these strains, although there are published data indicating that NDMs may also hydrolyze aztreonam [24,25].

Taking into account that at the time of the survey, the only “new entry” antibiotic available in the hospital was ceftazidime/avibactam, emphasis was given to the last resort drugs colistin, fosfomycin, and tigecycline [11].

Ceftazidime/avibactam, the only available antibiotic combination of a new β-lactam/β-lactamase inhibitor, was found to exhibit excellent activity in our survey against KPC- and OXA-48 producers, underscoring its value as a treatment for these infections.

Our results revealed that the increase in resistance to antibiotics such as ceftazidime/avibactam might reflect the increase in NDM isolates, as all the isolates that were resistant to ceftazidime/avibactam were MBL-positive. It may also reflect the entry of this new antibiotic in the Greek market.

The antimicrobial resistance trend we observed in this study revealed that colistin antimicrobial resistance rates fluctuated between 45.3%, 33.7%, 39.6%, and 43.2%. These findings indicate a statistically significant decrease in the resistance rate, likely reflecting the restrained use of colistin in our hospital due to the documented higher mortality attributed to its use [26,27]. Although colistin (polymyxin E) remains a potential treatment for carbapenem-resistant pathogens due to its broad-spectrum activity, its low efficacy as a monotherapy [28] and its potential toxicity (nephrotoxicity and neurotoxicity) limit its use [26,27].

As for fosfomycin (iv formulation), the resistance rate increased from 28.3% in 2021 to 59.9% in 2022 and to 75.9% in 2023, indicating that fosfomycin retains restrained potential against CR Enterobacterales. In 2024, though, EUCAST lowered the fosfomycin iv breakpoint to S ≤ 8 mg/L, R > 8 mg/L and now holds only for *E. coli* strains due to lack of evidence for other Enterobacterales. However, according to the WHO Essential Medicines List for optimal use—be AWaRe, fosfomycin belongs to “The Reserve group”. This group includes antibiotics that should be treated as last-resort options, indicating the appropriate use of antibiotics from clinicians and the optimization of antibiotic agents [29]. Tigecycline resistance exhibited a sharp increase to 40.7%, 96.7%, 95.8%, and 95.6% for each year studied.

The antimicrobial profile of Enterobacterales during the period studied, when stratified by carbapenemase type, indicated that KPC-positive isolates exhibited resistance rates to ceftazidime/avibactam, colistin, gentamicin, and fosfomycin (iv formulation) at rates of 0%, 34.9%, 62.9%, and 64.8%, respectively, emphasizing the excellent activity of ceftazidime/avibactam. Therefore, these agents may constitute potential treatment options in this context. As for MBLs, the situation is challenging, with no drug exhibiting excellent activity and resistance rates to fosfomycin, colistin, and gentamicin at percentages of 45.2%, 54.7%, and 70.5% for NDM and 66.2%, 58%, and 72.8% for VIM, respectively, indicating that these drugs may remain as treatment options to a certain extent. Aztreonam may also remain a choice for the treatment of VIM-producers, exhibiting a resistance rate of 63.4%. Regarding OXA-48-producing isolates, ceftazidime/avibactam, amikacin, and trimethoprim/sulfamethoxazole may constitute an appropriate therapeutic option, showing rates of resistance of 0%, 17.6%, and 36%, respectively, highlighting the excellent activity of ceftazidime/avibactam followed by amikacin. These agents, including trimethoprim/sulfamethoxazole, when appropriate, could be useful in the treatment of infections due to these isolates. Double combinations also presented challenging antimicrobial profiles. Regarding KPC/VIM, only colistin and fosfomycin demonstrated resistance rates lower that 85%, with percentages of 45.3% and 55.9%, respectively. As for KPC/NDM producers, the second most frequent combination encountered in our study, colistin, gentamicin, and nitrofurantoin, exhibited resistance rates with percentages of 32.5%, 52.6%, and 69.2%, respectively. Overall, these data show that metallo-β-lactamase-positive and especially NDM-positive isolates, as well as the dual-carbapenemase-positive isolates, were the most difficult to treat. Ceftazidime/avibactam seems to have changed the picture among KPC and OXA-48 producers. Notably, the OXA-48 producers in our study showed very good activity against amikacin as well.

The COVID-19 pandemic has led to excessive use of antibiotics along with diversion from the surveillance of antimicrobial resistance, factors that might have an impact in the increasing rate of antimicrobial resistance [6]. Notably, in our study, antimicrobial resistance rates for almost all antibiotics tested were significantly increased in the post COVID-19 period (2022 and 2023) compared to the first study year (2020) for almost all antibiotics, including those characterized as last-resort antibiotics. Moreover, it has been proposed that COVID-19 may facilitate the spread of CREs by promoting bacterial attachment and colonization in the respiratory tract, potentially leading to higher rates of CRE infections [30,31,32]. Significant variability exists in reported antimicrobial resistance trends due to the pandemic. Aligned with these data, our study showed alarming increases in the resistance rates of all antibiotics tested during the pandemic, further exacerbating the worsening situation of antimicrobial resistance in our country.

The observed fluctuations in carbapenem-resistant pathogens during the pandemic emphasize the need for ongoing surveillance and research to address antimicrobial resistance and develop effective treatment and infection control strategies. However, the introduction of new antibiotics mentioned above both alone and in combination with older regimens could contribute to the control of infections cause by CRE strains. The development of new, effective antimicrobial agents, especially those targeting pathogens with MBLs, remains a high priority. Comprehensive and continuous surveillance at all levels—local, national, regional, and global—is essential for the monitoring, guiding, and treatment of the changing epidemiology.

The limitations of our study include the fact that only the available antibiotics in our hospital were studied. New antimicrobial agents such as cefiderocol and the new β-lactam and β-lactamase inhibitor combinations such as meropenem/vaborvactam, imipenem-cilastatin/relebactam, and ceftozolane/tazobactam that were not widely introduced in Greece during the studied period were not included in this work. The above antibiotics may constitute additional therapeutic options against infections caused by CREs. Another limitations is that although NG-Test CARBA-5 (NG-Biotech) demonstrates exemplary performance in identifying the five most prevalent carbapenemases (100% sensitivity and 99.75% specificity for Enterobacterales using molecular identification as the gold standard), there is evidence that some variants (e.g., KPC-31-positive or KPC-114 -positive) with resistance to ceftazidime/avibactam are not detected using immunochromatography [33]. Additionally, our study is limited by its focus on a single hospital, although it is one of the largest in Athens, with many referrals from all over the country. Finally, another limitation is that the increasing trend in the incidence of CRE during the four-year period was studied on the basis that each isolated strain corresponded to a clinical infection and that no or insignificant numbers of cases were missed.

## 4. Materials and Methods

### 4.1. Study Design

This observational epidemiological study was conducted at the clinical laboratory of the tertiary care general hospital “Georgios Gennimatas”, Athens, one of the largest hospitals in the region of Attica. This study included all consecutive and non-repetitive single-patient CREs detected during 2020–2023. CREs were defined as Enterobacterales that exhibited resistance to at least one carbapenem (imipenem, meropenem, or ertapenem) based on the EUCAST criteria for minimum inhibitory concentration (MIC) interpretive breakpoints [34]. These clinical isolates were recovered from individual patients from all hospital departments (Orthopedic, Pathological, Surgical, Hematology, ICU, AED, Accident and Emergency Department—COVID, Nephrology/Urology, and others). They were collected from various sites, including blood, urine, wounds, bronchial secretions, and respiratory tract specimens, from individual patients hospitalized in different departments. This survey did not require patient consent documentation, as no personal data were used.

### 4.2. Bacterial Identification and Antimicrobial Susceptibility Testing

Identification of the clinical isolates was performed using the VITEK 2 system (bioMérieux, Marcy-l’Étoile, France), while antimicrobial susceptibility testing for a range of antibiotics, including ciprofloxacin, levofloxacin, piperacillin/tazobactam, cefepime, ceftazidime, ceftazidime/avibactam, aztreonam, imipenem, meropenem, gentamycin, tobramycin, amikacin, trimethoprim/sulfamethoxazole, fosfomycin (per os, iv formulation), colistin, and nitrofurantoin, was carried out using either the VITEK 2 system or the disk diffusion method and was interpreted based on EUCAST breakpoints published in the respective year.

As for tigecycline, the FDA breakpoints for Enterobacterales S ≤ 2 mg/L, I = 4 mg/L and R ≥ 8 mg/L were used. For fosfomycin, EUCAST clinical breakpoints for each year were used, i.e., fosfomycin IV for Enterobacterales S ≤ 32 mg/L, R > 32 mg/L. For oral fosfomycin, the clinical breakpoint of S ≥ 24 mm, R < 24 mm was applied to all Enterobacterales for urinary infections according to Clinical Breakpoint Tables v. 10.0 [35] for all years studied. However, from Clinical Breakpoints Tables v. 11.0 [36] onward, these zone diameter breakpoints hold only for *E.coli*. Nevertheless, they were still used for MDR isolates from other Enterobacterales upon clinician request, due to the habit formed by previous EUCAST versions that applied these breakpoints to all Enterobacterales. The reference method of agar dilution is laborious and was not set up in our laboratory, so VITEK and Kirby Bauer were mainly used. As for colistin, the sensitive isolates were confirmed with the broth microdilution method.

### 4.3. Detection of Carbapenemase Production

Detection and differentiation of the five most prevalent carbapenemases (KPC, VIM, IMP, NDM, and OXA-48) were performed using a lateral flow immunoassay (LFIA) (NG-Test CARBA5, NG Biotech, Guipry, France) and a FilmArray system (bioMérieux, Marcy l’Étoile, France) for blood cultures following the manufacturer’s guidelines.

### 4.4. Statistical Analysis

Statistical analysis was conducted using SPSS IBM version 25 (Released 2017; IBM Corp., Armonk, NY, USA). Categorical variables were analyzed using the chi-square test for the comparison of data between groups. Spearman’s correlation analysis was used to evaluate the association between the years and the total number of CPE infections per year. Statistical significance was set at a *p* value of 0.05.

## 5. Conclusions

Our study delineates the increase in infections attributed to carbepemase-producing isolates from 2020 to 2023. It demonstrates that CRE infections are mainly encountered as UTI infections, followed by systemic bloodstream infections, indicating that the main portal of entry for these infections is the urinary system, obviously associated with urinary catheter use in hospitals. KPC remains the predominant carbapenemase in our setting, followed by the NDM, VIM, and OXA-48 carbapenemases. Interestingly, the percentage of double carbapenemase-producing isolates is not negligible. NDM is gaining ground versus VIM, but still, MBL-positive isolates significantly increased during the study period due to the dissemination of NDM carbapenemase. Taking into account that the discovery of new drugs for class B carbapenemases is lagging, it is likely that the MBLs may take over the situation in the near future. It is also evident that antimicrobial resistance is more alarming than ever, with the exception of a few options in some cases, delineating the impact of the pandemic on antimicrobial resistance. Our study highlights the need for broader research and underscores the urgent requirement for new antimicrobial agents and enhanced surveillance to monitor the evolving epidemiology of carbapenemases.

## Figures and Tables

**Table 1:** Total number of patients infected with CPEs during the study.

	2020	2021	2022	2023
Male	255 (64.4%)	257 (58.3%)	259 (47%)	358 (56.6%)
Female	141 (35.6%)	184 (41.7%)	292 (53%)	275 (43.4%)
	*n* = 396	*n* = 441	*n* = 551	*n* = 633

**Table 2:** Distribution of clinical isolates by sample type across the study years.

Type of Sample (%)	2020	2021	2022	2023
Blood culture	55 (13.9%)	75 (17%)	84 (15.2%)	48 (7.6%)
Bronchial secretions	36 (9.1%)	61 (13.8%)	62 (11.3%)	25 (3.9%)
CVC (Central Venous Catheter)	28 (7.1%)	26 (5.9%)	19 (3.4%)	25 (3.9%)
Urine	157 (39.6%)	174 (39.5%)	262 (47.5%)	284 (44.9%)
Wound specimen	52 (13.1%)	38 (8.6%)	26 (4.7%)	0 (0%)
Multiple sample areas	68 (17.2%)	66 (15%)	69 (12.5%)	161 (25.4%)
Other	0 (0%)	1 (0.2%)	29 (5.3%)	90 (14.2%)
Total	*n* = 396	*n* = 441	*n* = 551	*n* = 633

**Table 3:** Total number of species isolated per year.

	2020	2021	2022	2023	2020–2023
***Klebsiella*** **spp.**	355 (89.6%)	415 (94.1%)	494 (89.7%)	571 (90.2%)	1835 (90.9%)
***E. coli***	11 (2.8%)	8 (1.8%)	6 (1.1.%)	17 (2.7%)	42 (2.1%)
***Enterobacter*** **spp.**	12 (3.0%)	6 (1.4%)	10 (1.8%)	7 (1.1.%)	35 (1.8%)
***P. mirabilis***	9 (2.3%)	7 (1.6%)	12 (2.2.%)	10 (1.6%)	38 (1.9%)
***P. stuartii***	5 (1.3%)	5 (1.1%)	23 (4.2%)	21 (3.3%)	54 (2.5%)
***Serratia*** **spp.**	2 (0.5%)	-	3 (0.5%)	3 (0.5%)	8 (0.4%)
***Citrobacter*** **spp.**	2 (0.5%)	-	2 (0.4%)	1 (0.2%)	5 (0.3%)
***Morganella*** **spp.**	-	-	1 (0.2%)	3 (0.5%)	4 (0.2%)
**Total**	*n* = 396	*n* = 441	*n* = 551	*n* = 633	*n* = 2021

**Table 4:** Total number of Enterobacterales isolated in the ICU.

		2020	2021	2022	2023
**Enterobacterales**	*Klebsiella* spp.	82/355	129/406	87/494	92/562
	*Enterobacter* spp.	2/12	-	2/10	
	*E. coli*	-	-	1/6	1/17
	*P. mirabilis*	6/9	-	2/12	1/10
	*P. stuartii*	-	-	13/23	3/21
	*Morganella* spp.	-	-	1/1	-
	Total	90/396 22.7%	129/441 29.3%	106/551 19.2%	97/633 15.3%

**Table 5:** Total percentage (%) of each carbapenemase type among Enterobacterales/year.

	2020	2021	2022	2023
**KPC**	161/396 40.7%	176/441 39.9%	295/551 53.5%	289/633 45.7%
**VIM**	61/396 15.4%	39/441 8.8%	71/551 12.9%	75/633 11.8%
**NDM**	66/396 16.7%	113/441 25.6%	129/551 23.4%	167/633 26.4%
**OXA**	42/396 10.6%	5/441 1.1%	1/551 0.2%	3/633 0.5%
**KPC/OXA**	3/396 0.8%	1//441 0.2%	0/551 0.0%	0/633 0.0%
**KPC/VIM**	50/396 12.6%	68/441 15.4%	40/551 7.3%	68/633 10.7%
**OXA/NDM**	7/396 1.8%	0/441 0.0%	2/551 0.4%	1/633 0.2%
**VIM/NDM**	2/396 0.5%	2/441 0.5%	2/551 0.4%	2/633 0.3%
**KPC/NDM**	3/396 0.8%	37/441 8.4%	11/551 2.0%	27/633 4.3%
**OXA/VIM**	0/396 0.0%	0/441 0.0%	0/551 0.0%	1/633 0.2%
**KPC/OXA/NDM**	1/396 0.3%	0/441 0.0%	0/551 0.0%	0/633 0.0%

**Table 6:** Distribution of carbapenemase type per species and year. The number of positive isolates for each carbapenemase/total number of each species for each year studied are presented.

			2020	2021	2022	2023
**KPC**	*Klebsiella* spp.		155/355	171/419	290/494	281/571
	Other Enterobacterales	*E. coli*	5/11	4/8	3/6	8/17
		*Enterobacter* spp.	1//12	1/6	1/10	0/7
		*P. stuartii*	0/5	0/5	1/23	0/21
**VIM**	*Klebsiella* spp.		34/355	24/419	25/494	35/571
	Other Enterobacterales	*E. coli*	2/11	2/8	3/6	2/17
		*Enterobacter* spp.	10/12	2/6	6/10	5/7
		*P. mirabilis*	9/9	6/7	11/12	8/10
		*P. stuartii*	4/5	5/5	22/23	21/21
		*Serratia* spp.	2/2	-	2/3	3/3
		*Citrobacter* spp.	-	-	1/2	-
		*Morganella* spp.	-	-	1/1	1/3
**NDM**	*Klebsiella* spp.		63/355	109/415	127/494	157/571
	Other Enterobacterales	*E. coli*	2/11	1/8	0/6	5/17
		*Enterobacter* spp.	1/12	3/6	1/10	1/7
		*P. mirabilis*	0/9	0/7	1/12	1/10
		*Citrobacter* spp.	0/2	0/2	0/2	1/1
		*Morganella* spp.	-	-	0/1	2/3
**OXA**	*Klebsiella spp.*		40/355	5/415	1/494	2/571
	Other Enterobacterales	*E. coli*	2/11	0/8	0/6	0/17
		*P. mirabilis*	0/9	0/7	0/12	1/10
**KPC/OXA**	*Klebsiella spp.*		3/355	1/415	-	-
	Other Enterobacterales		-	-	-	-
**KPC/VIM**	*Klebsiella* spp.		49/355	67/415	38/494	66/571
	Other Enterobacterales	*Citrobacter* spp.	1/2	-	-	0/1
		*E. coli*	0/11	1/8	0/6	2/17
		*Enterobacter* spp.	0/12	0/6	2/10	0/7
**OXA/NDM**	*Klebsiella* spp.		7/355	-	2/494	1/571
	Other Enterobacterales			-	-	-
**VIM/NDM**	*Klebsiella* spp.		1/355	1/415	-	2//571
	Other Enterobacterales	*P. stuartii*	1/5	0/5	0/23	0/21
		*P. mirabilis*	0/9	1/7	0/12	0/10
		*Citrobacter* spp.	0/2	-	1/2	0/1
		*Serratia* spp.	0/2	-	1/3	0/3
**KPC/NDM**	*Klebsiella* spp.		2/355	37/415	11/494	27/571
	Other Enterobacterales	*Citrobacter* spp.	1/2	-	0/2	0/1
**OXA/VIM**	*Klebsiella* spp.		0/355	0/415	0/494	0/571
	Other Enterobacterales	*Enterobacter* spp.	0/12	0/6	0/10	1/7
**KPC/OXA/NDMM**	*Klebsiella* spp.		1/355	0/415	0/494	0/571
	Other Enterobacterales		-	-	-	-
Total			*n* = 396	*n* = 441	*n* = 551	*n* = 633

**Table 7:** Antimicrobial resistance trends in *Klebsiella* spp. (2020–2023).

Antibiotic	Resistance	2020 vs. 2021	2021 vs. 2022	2022 vs. 2023	2020 vs. 2023
Piperacillin/Tazobactam	2020—324/351 (92.3%)	*p*-value ≤ 0.00001	NS	NS	*p*-value ≤ 0.00001
	2021—412/415 (99.3%)				
	2022—492/494 (99.6%)				
	2023—570/570 (100%)				
Ceftazidime	2020—349/355 (98.3%)	*p*-value = 0.034687	NS	NS	*p*-value = 0.009656
	2021—414/145 (99.8%)				
	2022—493/494 (99.8%)				
	2023—571/571 (100%)				
Cefepime	2020—315/355 (88.7%)	*p*-value ≤ 0.00001	NS	*p*-value = 0.038303	*p*-value ≤ 0.00001
	2021—405/415 (97.6%)				
	2022—479/494 (97.0%)				
	2023—564/571 (98.8%)				
Aztreonam	2020—311/355 (87.6%)	*p*-value = 0.002035	NS	NS	*p*-value = 0.000059
	2021—390/415 (94.0%)				
	2022—473/494 (95.7%)				
	2023—542/571 (94.9%)				
Imipenem	2020—352/355 (99.2%)	NS	NS	NS	NS
	2021—414/415 (99.8%)				
	2022—494/494 (100%)				
	2023—569/571 (99.6%)				
Meropenem	2020—350/355 (98.6%)	NS	NS	NS	NS
	2021—410/415 (98.8%)				
	2022—494/494 (100%)				
	2023—567/571 (99.3%)				
Gentamicin	2020—154/355 (43.4%)	*p*-value ≤ 0.00001	NS	NS	*p*-value =< 0.00001
	2021—300/415 (72.3%)				
	2022—361/493 (73.2%)				
	2023—421/571 (73.7%)				
Tobramycin	2020—314/354 (88.7%)	*p*-value = 0.003713	NS	NS	*p*-value = 0.001378
	2021—392/415 (94.5%)				
	2022—447/487 (91.8%)				
	2023—367/386 (95.1%)				
Amikacin	2020—173/354 (48.9%)	*p*-value ≤ 0.00001	NS	NS	*p*-value ≤ 0.00001
	2021—344/415 (82.9%)				
	2022—392/493 (79.5%)				
	2023—466/571 (81.6%)				
Ciprofloxacin	2020—331/349 (94.8%)	NS	NS	NS	*p*-value = 0.000417
	2021—403/415 (97.1%)				
	2022—482/493 (97.8%)				
	2023—558/565 (98.8%)				
Levofloxacin	2020—320/340 (94.1%)	NS	NS	NS	*p*-value = 0.008241
	2021—399/415 (96.1%)				
	2022—478/493 (97.0%)				
	2023—557/571 (97.5%)				
Trimethoprin/Sulfamethoxazole	2020—254/354 (71.8%)	*p*-value ≤ 0.00001	NS	*p*-value = 0.010091	*p*-value ≤ 0.00001
	2021—355/415 (85.5%)				
	2022—403/485 (83.1%)				
	2023—505/570 (88.6%)				
Colistin	2020—160/353 (45.3%)	*p*-value = 0.001034	NS	*p*-value ≤ 0.00001	*p*-value ≤ 0.00001
	2021—140/415 (33.7%)				
	2022—195/493 (39.6%)				
	2023—245/257 (43.2%)				
Tigecycline	2020—68/167 (40.7%)	*p*-value =< 0.00001	NS	NS	*p*-value =< 0.00001
	2021—295/305 (96.7%)				
	2022—346/361 (95.8%)				
	2023—371/388 (95.6%)				
Ceftazidime/Avibactam	2020—11/179 (6.1%)	NS	*p*-value ≤ 0.00001	*p*-value ≤ 0.00001.	*p*-value ≤ 0.00001
	2021—14/168 (8.3%)				
	2022—120/374 (32.1%)				
	2023—274/551 (49.7%)				
Fosfomycin (per os)	2020—6/29 (20.7%)	*p*-value = 0.000458	NS	*p*-value = 0.02551	*p*-value ≤ 0.00001
	2021—8/8 (100%)				
	2022—54/66 (81.8%)				
	2023—131/142 (92.3%)				
Fosfomycin (iv)	2020—0/0 (0%)		*p*-value ≤ 0.00001	*p*-value = 0.000238	
	2021—36/127 (28.3%)				
	2022—112/187 (59.9%)				
	2023—211/278 (75.9%)				
Nitrofurantoin	2020—104/128 (81.3%)	*p*-value = 0.005559	NS	NS	*p*-value ≤ 0.00001
	2021—92/98 (93.9%)				
	2022—188/198 (94.9%)				
	2023—184/188 (97.9%)				

**Table 8:** Antimicrobial resistance to the main antibiotics among all Enterobacterales stratified by the type of carbapenemase during the four-year period studied.

Antimicrobial	KPC	NDM	VIM	OXA	KPC/OXA	KPC/VIM	OXA/NDM	VIM/NDM	KPC/NDM	OXA/VIM	KPC/OXA/NDM
Piperacillin/Tazobactam	903/919 (98.3%)	468/475 (98.5%)	228/245 (93.1%)	49/51 (96.1%)	4/4 (100%)	222/225 (98.7%)	8/9 (88.9%)	8/8 (100%)	78/78 (100%)	1/1 (100%)	1/1 (100%)
Ceftazidime	917/921 (99.6%)	474/475 (99.8%)	243/245 (99.2%)	50/51 (98%)	4/4 (100%)	226/226 (100%)	10/10 (100%)	8/8 (100%)	78/78 (100%)	1/1 (100%)	1/1 (100%)
Cefepime	891/921 (96.7%)	475/475 (100%)	239/246 (97.2%)	51/51 (100%)	4/4 (100%)	224/226 (99.1%)	10/10 (100%)	8/8 (100%)	78/78 (100%)	1/1 (100%)	1/1 (100%)
Aztreonam	920/921 (99.9%)	419/475 (88.2%)	156/246 (63.4%)	49/51 (96.1%)	4/4 (100%)	221/226 (97.8%)	7/10 (70%)	5/8 (62.5%)	78/78 (100%)	1/1 (100%)	1/1 (100%)
Gentamicin	579/921 (62.9%)	335/475 (70.5%)	179/246 (72.8%)	42/51 (82.4%)	2/4 (50%)	193/225 (85.8%)	6/10 (60%)	5/8 (62.5%)	41/78 (52.6%)	1/1 (100%)	1/1 (100%)
Tobramycin	724/828 (87.4%)	398/409 (97.3%)	222/226 (98.2%)	44/47 (93.6%)	4/4 (100%)	206/206 (100%)	9/10 (90%)	7/7 (100%)	65/66 (98.5%)		1/1 (100%)
Amikacin	698/920 (75.9%)	359/475 (75.6%)	192/246 (78%)	9/51 (17.6%)	2/4 (50%)	204/225 (90.7%)	8/10 (80%)	7/8 (87.5%)	71/78 (91%)	1/1 (100%)	0/1 (0%)
Ciprofloxacin	883/915 (96.5%)	463/471 (98.3%)	229/243 (94.2%)	48/50 (96%)	4/4 (100%)	222/224 (99.1%)	10/10 (100%)	6/8 (75%)	78/78 (100%)	1/1 (100%)	1/1 (100%)
Levofloxacin	884/917 (96.4%)	463/472 (98.1%)	232/245 (94.7%)	47/48 (97.9%)	4/4 (100%)	221/223 (99.1%)	10/10 (100%)	6/8 (75%)	78/78 (100%)	1/1 (100%)	1/1 (100%)
Trimethoprin/Sulfametho	628/916 (68.6%)	394/475 (82.9%)	234/241 (97.1%)	18/50 (36%)	2/4 (50%)	225/225 (100%)	4/10 (40%)	7/8 (87.5%)	73/78 (93.6%)	1/1 (100%)	0/1 (0%)
Colistin	320/917 (34.9%)	260/475 (54.7%)	142/245 (58%)	41/50 (82%)	2/4 (50%)	102/225 (45.3%)	6/10 (60%)	7/8 (87.5%)	25/77 (32.5%)	0/1 (0%)	1/1 (100%)
Tigecycline	552/640 (86.3%)	299/339 (88.2%)	133/154 (86.4%)	19/22 (86.4%)	1/2 (50%)	149/157 (94.9%)	5/5 (100%)	4/5 (80%)	55/58 (94.8%)		1/1 (100%)
Ceftazidime/Avibactam	0/729 (0%)	243/245 (99.2%)	116/117 (99.1%)	0/43 (0%)	0/4 (0%)	95/96 (99%)	4/4 (100%)	4/4 (100%)	36/36 (100%)	1/1 (100%)	
Fosfomycin (iv)	177/273 (64.8%)	94/208 (45.2%)	45/68 (66.2%)	3/4 (75%)	1/1 (100%)	33/59 (55.9%)	1/2 (50%)	0/4 (0%)	13/16 (81.3%)	0/1 (0%)	
Nitrofurantoin	246/270 (91.1%)	167/181 (92.3%)	65/74 (87.8%)	15/15 (100%)	2/2 (100%)	46/50 (92%)	4/4 (100%)	3/3 (100%)	9/13 (69.2%)		

## Data Availability

The datasets used and/or analyzed during the current study are available in the article. Any further information is available from the corresponding author (V.K.) upon reasonable request.
